# Supplementary figures and images for: Correlates of hybridization in plants
Source: Evol Lett. 2019 Oct 28;3(6):570–85. doi: 10.1002/evl3.146 (PMC6906982; doi:10.1002/evl3.146)

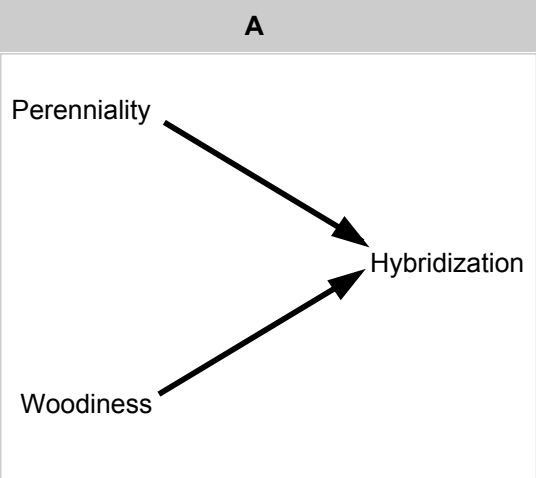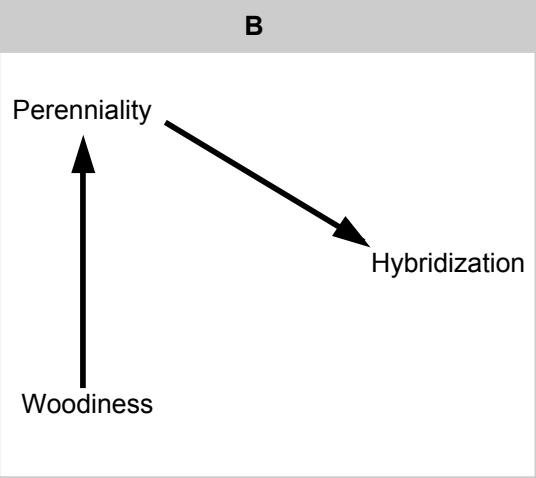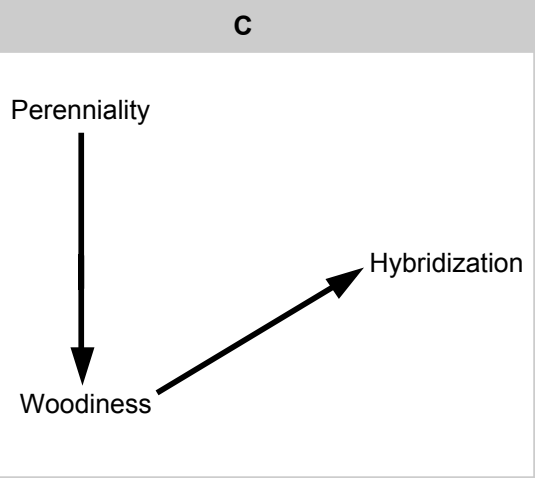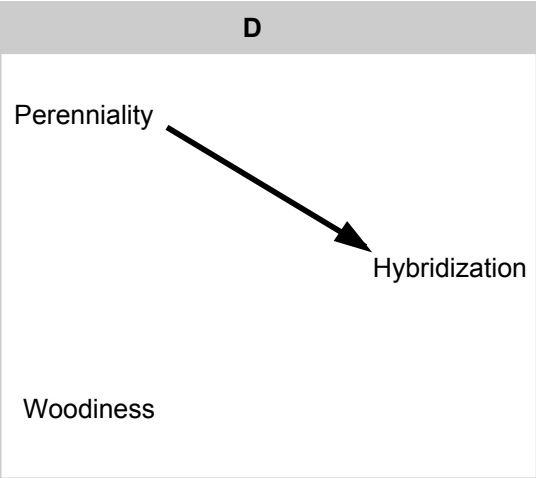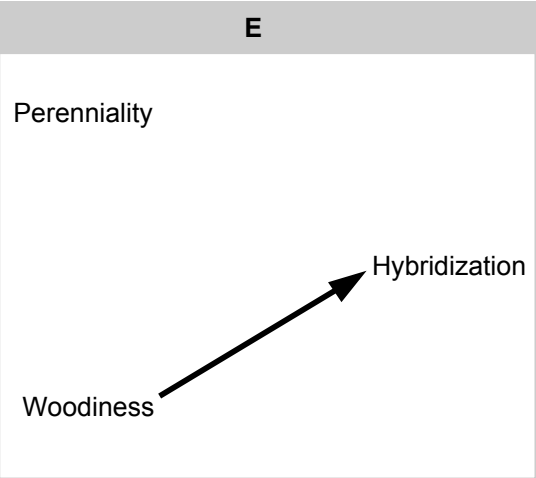

Supplement: Supplementary file 1 — Figure S1. [file EVL3-3-570-s001.pdf]
